# New Antibiotic Molecules: Bypassing the Membrane Barrier of Gram Negative Bacteria Increases the Activity of Peptide Deformylase Inhibitors

**DOI:** 10.1371/journal.pone.0006443

**Published:** 2009-07-30

**Authors:** Laurent Mamelli, Sylvain Petit, Jacqueline Chevalier, Carmela Giglione, Aurélie Lieutaud, Thierry Meinnel, Isabelle Artaud, Jean-Marie Pagès

**Affiliations:** 1 UMR-MD1, Transporteurs Membranaires, Chimiorésistance et Drug-Design, Facultés de Médecine et de Pharmacie, IFR 88, Université de la Méditerranée, Marseille, France; 2 UMR8601-CNRS, Université Paris Descartes, Laboratoire de Chimie et Biochimie, Pharmacologiques et Toxicologiques, Paris, France; 3 UPR2355-CNRS, Institut des Sciences du Végétal, Centre National de Recherche Scientifique, Gif sur Yvette, France; Charité-Universitätsmedizin Berlin, Germany

## Abstract

**Background:**

Multi-drug resistant (MDR) bacteria have become a major concern in hospitals worldwide and urgently require the development of new antibacterial molecules. Peptide deformylase is an intracellular target now well-recognized for the design of new antibiotics. The bacterial susceptibility to such a cytoplasmic target primarily depends on the capacity of the compound to reach and accumulate in the cytosol.

**Methodology/Principal Findings:**

To determine the respective involvement of penetration (influx) and pumping out (efflux) mechanisms to peptide deformylase inhibitors (PDF-I) activity, the potency of various series was determined using various genetic contexts (efflux overproducers or efflux-deleted strains) and membrane permeabilizers. Depending on the structure of the tested molecules, two behaviors could be observed: (i) for actinonin the first PDF-I characterized, the AcrAB efflux system was the main parameter involved in the bacterial susceptibility, and (ii), for the lastest PDF-Is such as the derivatives of 2-(5-bromo-1*H*-indol-3-yl)-*N*-hydroxyacetamide, the penetration through the membrane was a important limiting step.

**Conclusions/Significance:**

Our results clearly show that the bacterial membrane plays a key role in modulating the antibacterial activity of PDF-Is. The bacterial susceptibility for these new antibacterial molecules can be improved by two unrelated ways in MDR strains: by collapsing the Acr efflux activity or by increasing the uptake rate through the bacterial membrane. The efficiency of the second method is associated with the nature of the compound.

## Introduction

Multi-drug resistance (MDR) in Gram-negative bacteria is frequently reported in clinical isolates [1], [2]. This strongly limits therapeutic options and is a major cause of mortality in hospital acquired infections [1]–[4]. MDR is prevalent in important Gram-negative clinical pathogens such as *Escherichia coli, Salmonella* spp., *Klebsiella* spp., *Enterobacter* spp., and *Pseudomonas* spp. In these major pathogens, three major bacterial strategies are involved in the development of drug resistance: 1) the membrane barrier (acting to limit the required intracellular dose of an antibiotic), 2) the enzymatic barrier (producing detoxifying enzymes that degrade or modify the antibiotic), 3) the target protection barrier (mutation or expression of a molecule that impairs target recognition and thus antimicrobial activity) [5]. These Gram-negative bacteria, responsible for a large portion of antibiotic-resistant bacterial diseases, display a complex cell envelope comprising an outer membrane and an inner membrane delimiting the periplasm [6]. The outer membrane contains various protein channels which are involved in the transport of various compounds including several classes of antibiotics [6], [7]. Bacterial adaptation to reduce the outer membrane permeability is an increasing problem worldwide, which contributes, along with efflux systems, to the emergence and dissemination of antibiotic resistance. Consequently, it is important to explore the activity of existing and new antibiotic compounds by using different bacterial strains harbouring various resistance backgrounds and in the presence of diverse chemicals recently described as potent inhibitors of resistance mechanism or facilitator of antibiotic activity [8]–[10].

Face to this continuous emerging threat, several novel bacterial targets have been described as an alternate therapeutic solution to the emergence and dissemination of MDR bacterial isolates [11], [12]. Peptide deformylase (PDF) is involved in the cleavage of the *N*-formyl group of nascent polypeptide. This process is essential to the growth of bacteria [13] and represents a novel attractive target for new antibacterial molecules [14]–[16]. Peptide deformylase inhibitors (PDF-Is) have been characterized and described to be active in various bacterial pathogens. Most of them such as actinonin, the main characterized inhibitor, are pseudopeptidic molecule [17]. Recently we described a new series of heterocyclic compounds, with an indol scaffold, that inhibit efficiently bacterial PDF in the nM range [18], [19]. Two main mechanisms mediating resistance to peptide deformylase inhibitors in bacteria have been previously described. The first is amino acid substitutions within the target protein (Def) , and the second is the formylation bypass which results from mutational loss of methionyl tRNA formyltransferase (Fmt) or of the *folD* gene [20]–[22]. However a discrepancy of activity was observed with the efflux system that seriously compromised the PDF-Is action in some efflux producing strains [23], [24]. The role of AcrB and TolC component of efflux pump has been reported in the susceptibility of *E. coli* and *Haemophilus influenzae* [18], [23].

**Figure 1:**
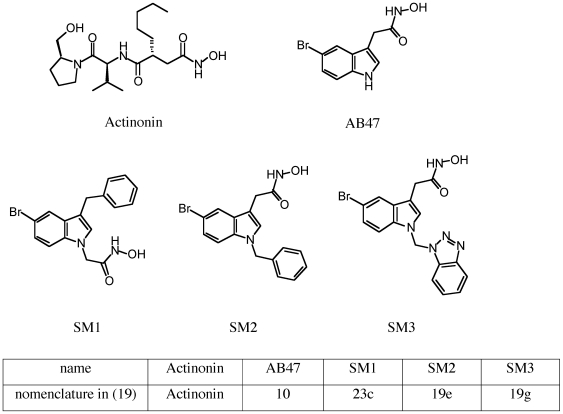
Structures of PDF-Is used.

The MDR phenotype is often linked to with a general alteration of membrane properties including a decrease in membrane permeability associated with an overexpression of efflux pumps [5]. We decided to investigate the involvement of the membrane barrier (the “in and the out” transport) in the activity of some indolic PDF-Is structurally unrelated to actinonin group (Figure 1) and recently produced [18], [19] in various strains and clinical MDR isolates.

## Results

### Activity of new PDF-Is and membrane permeability

Actinonin was used as reference molecule to characterize the antibacterial activities of the newly produced PDF-Is for which an *in vitro* activity has been previously reported [19]. In Table 1 were presented the results obtained on isogenic strains in the absence or in the presence of various sub-inhibitory concentrations of the cyclic peptide antibiotic polymyxin B (Pol B) or its derivative the polymyxin nonapeptide (PMBN) known to increase membrane permeability [25], [26].

**Table 1:** Determination of antibacterial activity of various PDF-Is on *E. coli* strains.

Strains	Actinonin MIC (µg/ml)			AB47 MIC (µg/ml)			SM1 MIC (µg/ml)			SM2 MIC (µg/ml)			Norfloxacin MIC (µg/ml)			Chloramphenicol MIC (µg/ml)		
Permea^a^	0	PMBN^b^	Pol B^c^	0	PMBN^a^	Pol B^b^	0	PMBN^a^	Pol B^b^	0	PMBN^a^	Pol B^b^	0	PMBN^a^	Pol B^b^	0	PMBN^a^	Pol B^b^
AG100	128	2 (4)	16 (128)	512	8 (32)	64 (128)	>512	4 (8)	>128 (>128)	>512	8 (16)	128 (>128)	0.25	0.125	0.125	8	2	4
AG102	128	16	128	512	32	128	>512	16	>128	>512	16	128	1	0.5	1	16	16	16
AG100A	1	0.125 (0.25)	0.25 (0.25)	64	16 (16)	32 (32)	128	4 (4)	16 (32)	64	8 (8)	32 (32)	0.06	0.06	0.03	1	1	0.5
AG100Atet	512	16	>128	256	16	16	>512	16	>128	>512	8	>128	1	0.5	1	64	32	64

Values are means of four independent assays.

a permeabilizer used.

b PMBN was used at 1/5 MIC, in bracket the results with 1/10 MIC are indicated.

c Pol B was used at 1/5 MIC, in bracket the results with 1/10 MIC are indicated.

In the absence of membrane permeabilizers, we observed a 128 fold decrease of actinonin MIC in the *acrAB* deleted strain compared to the parental ones. This suggests that AcrB pump is directly involved in the resistance observed in the parental strain towards this molecule. Regarding the other PDF-Is, we did not observed a susceptibility level similar to that obtained with actinonin. These results indicate that SM1, SM2, and AB47 compounds are not recognized as specific substrate for AcrAB efflux transporter or that another rate-limiting step is involved. Concerning SM3, whatever the tested strains or the conditions used, no antibacterial activity was detected (data not shown).

The effect of membrane permeabilizer, *e.g.* Pol B and PMBN, was assayed on the PDF-Is activities. The MICs for Pol B and PMBN were determined for each bacterial strain. From the respective MICs, a sub-inhibitory amount (MIC/5 and MIC/10) was added in the presence of each PDF-I. For actinonin, the presence of PMBN induced a serious decrease of MIC to the susceptible level whatever the strain tested. In the *acrAB* deleted strain, a small increase of susceptibility was noted (MIC of 0.25 µg/ml). Concerning the other molecules, the addition of PMBN, and Pol B at a lesser extent, induced a noticeable increase of susceptibility (Table 1). It is interesting to note that for SM1, SM2 and AB-47 whatever the strain background tested an important MIC decrease was induced in the presence of PMBN. Concerning SM3, no increase in the susceptibility was observed in the *acrAB* deleted strain in the absence or in presence of PMBN (data not shown). In addition, in the same conditions, presence of Pol B or PMBN, only a very limited effect was noted on the activity of usual antibiotics such as norfloxacin and chloramphenicol (Table 1).

The activity of the different PDF-Is was tested on other Gram-negative bacteria involved in human infectious diseases such as *Pseudomonas aeruginosa*, *Enterobacter aerogenes and Klebsiella pneumoniae* (Table 2). SM1 and actinonin exhibited no antibacterial activity on the *P. aeruginosa* strains including reference strain PA01 and clinical isolate 124. The addition of PMBN during the incubation noticeably increased the activity of actinonin, AB-47, SM1 (Table 2). A less efficient effect was obtained with SM2 (data not shown). It is interesting to note that the level of resistance for the *P. aeruginosa* clinical isolate (124 in table 2) which is a MDR strain with several mechanisms including efflux pump overexpression, was more significant for usual antibiotics (norfloxacin, tetracycline, etc).

**Table 2:** Activity of PDF-Is on MDR Gram-negative isolates.

	*P. aeruginosa*		*E. aerogenes*						*K. pneumoniae*		
	PA01	124	ATCC 13048	EA5	EA27	EA289	EA298	EA294	ATCC 11296	KP55	KP63
Actinonin	128	128	>128	128	128	>128	32	128	128	128	128/64
Actinonin + PMBN a	4	16	8	64	64	32	0.5	2	32	32	32
SM1	128	128	>128	>128	>128	>128	64	128	>128	>128	128
SM1 + PMBN a	2	8	8	32	32	32	8	16	64	64	128–64
AB47	128	>128	>128	>128	>128	256	128	128	128	128	128
AB47 + PMBN a	4	8	32	128	128	128	16	32	64	64	64–32
CM	256	256	8	>128	>128	>128	32 (16) b	32	16 (4) b	256	>512
NFX	2	64	0,5	128	128	128	16 (32) b	32	1 (0.5) b	16	8
CAZ	8	32	1	>512	>512	>512	>512	>512	1	>512	>512
ERY	1024	1024	512	512	512	512	32	128	128	512	512
TC	16	64	4	4	16	8	0.5(0.25) b	1	2 (2) b	>128	8

Values are means of four independent assays listed in µg/ml.

CM, chloramphenicol; NFX, norfloxacin; CAZ, ceftazidime; ERY, erythromycin; TC, tetracycline.

a PMBN was used at 1/5 MIC.

b in bracket the results obtained with 1/5 PMBN are indicated.

We observed similar profiles with other Gram-negative bacterial pathogens and the activity of actinonin was noticeably increased in the presence of membrane permeabilizer. The tested clinical MDR isolates (*E. aerogenes* EA5 and EA27; *K. pneumoniae* KP55 and KP63) exhibited a lower susceptibity towards other PDF-Is assayed under the same conditions. Moreover, in the derivative strains of MDR *E. aerogenes* clinical isolate EA27, we observed a significant role of the efflux pump: when the *tolC* or *acrAB* genes were knocked out, the presence of PMBN induced a noticeable increase of actinonin and SM1 activity, *e.g.* compare EA289 and EA298 strains in Table 2. Under these conditions, it is worthy of note that the *tolC* deletion was more efficient compare to *acrA* for actinonin (MICs of 0.5 and 2 respectively). A similar level was reached in the two *acrA* and *tolC* deleted strains for SM1 and AB47 (8 and 16, 16 and 32 respectively). It has been previously demonstrated that at least two distinct efflux pumps are active, AcrAB-TolC and another unidentified pump, in the EA27 clinical isolate and its EA289 derivative strain [27]. In these MDR isolates, the results indicated that the AcrAB-TolC could be the main transporter involved for actinonin efflux. By contrast, regarding SM1 and AB-47, additional pump may also modulate the activity of these molecules since in EA298 with PMBN the MICs was 8 and 16 compare to actinonin MIC (0.5). It is interesting to note that, in general,the new antibacterial molecules tested here seemed to be more efficient against clinical isolates compare to several hospital-used antibiotics (*e.g.*, erythromycin, ceftazidime).

### Effect of efflux pump inhibitors on PDF-Is activity

The impact of efflux pumps in antibiotic susceptibility has been largely demonstrated [28]–[30]. The results shown in Table 1 suggested an involvement of AcrAB pump in the actinonin efflux and at lesser extent for the other tested PDF-Is. In this context, to get more insight in the process we determine the effect of a well-described efflux pump inhibitor, PAβN which is able to block the activity of various efflux pumps in addition to AcrAB-TolC [8], [9]. In Table 3, the results indicated that the PAβN induced only a limited increase of PDF-Is activities compared to PMBN. Moreover, when the two molecules, PMBN and PAβN, were conjointly added no cumulative effect was observed (data not shown). Since AcrAB was involved in modulating actinonin activity, these results suggest that PAβN was not an effective competitive substrate for efflux of PDF-Is. In other words, the affinity constant of efflux pump for PDF-Is may be stronger compare to PAβN.

**Table 3:** Effect of efflux pump inhibitors and membrane permeabilizers.

Bacteria	Actinonin			AB47		
*E. coli*	0	PMBN^a^	PAβN	0	PMBN^a^	PAβN
AG100	128	2	32	512	8	128
AG102	128	16	64	512	32	128
AG100A	1	0.125	0.5	64	16	16
AG100Atet	512	8	32	256	16	128
*P. aeruginosa*						
124	128	16	32	>128	8	128

	SM1			SM2		
*E. coli*	0	PMBN^a^	PAβN	0	PMBN^a^	PAβN
AG100	>512	4	128	>512	8	128
AG102	>512	4	128	>512	16	128
AG100A	128	4	16	64	8	32
AG100Atet	>512	16	64	>512	8	64
*P. aeruginosa*						
124	128	8	128	>128	64	32

Values are means of four independent assays.

a PMBN was used at 1/5 MIC.

b PAβN was used at 20 µg/ml.

Same results were obtained when the assays were performed on *P. aeruginosa* strain (Table 3). We have similarly determined the effect of other inhibitors such as reserpine, verapamil and omeprazol. No significant change in the MICs of PDF-Is was obtained in the various *E. coli* strains (data not shown).

### Activity of PDF-Is on lipopolysaccharide (LPS) deep rough mutants, effect of detergents and chelators

**Figure 2:**
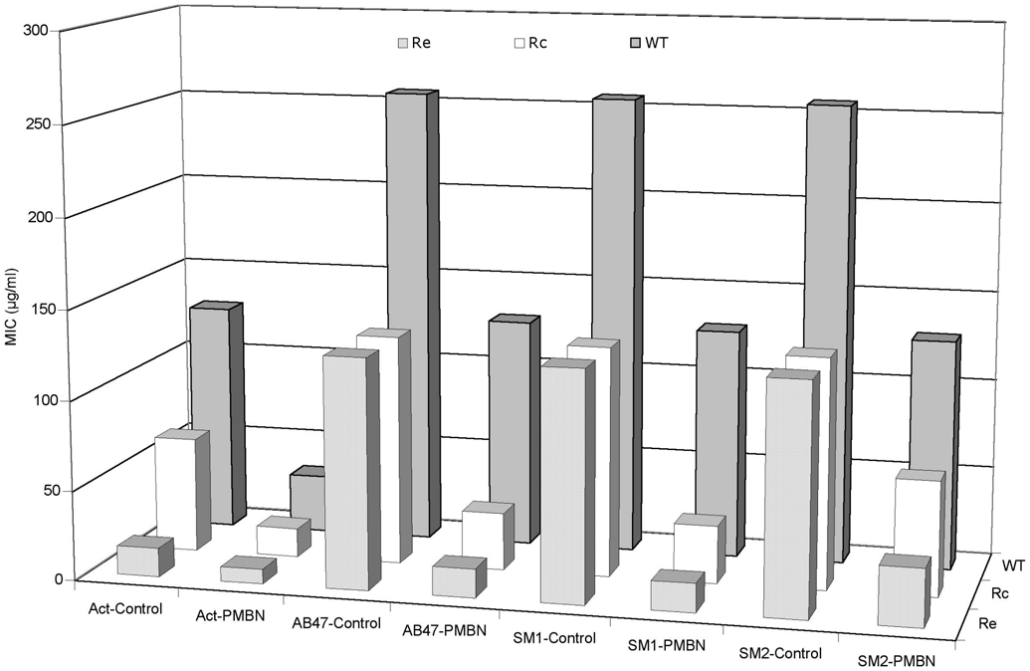
Activity of PDF-Is on *Salmonella* wild-type strain SL696 and its LPS-deficient mutants. Various isogenic strains, WT (intact LPS), Rc (truncated LPS: lipidA-KDO-hep-hep-Glc) and Re (truncated LPS: lipidA-KDO) previously described [31] were used. Act-Control, actinonin alone; Act-PMBN, actinonin + PMBN; AB47-Control, AB47 alone; AB47-PMBN, AB47 + PMBN; SM1-Control, SM1 alone; SM1-PMBN, SM1 + PMBN; SM2-Control, SM2 alone; SM2-PMBN, SM2 + PMBN. PMBN was used at 1/5 MIC. MIC values are in µg/ml.

In Gram negative bacteria, the LPS constitutes the outer leaflet of outer membrane and may impair the penetration of antibacterial agents [6]. Since we have observed that the addition of membrane permeabilizer such as Pol B or PMBN, are capable to induce a serious decrease of MICs, we tested a series of *Salmonella* typhimurium LT2 mutants producing truncated LPS [31] to assess a putative role of LPS structure in the PDF-Is activity. These strains have been previously used as standard strains to assess the role of LPS on the level of diffusion through the outer membrane [31]. As shown in Figure 2, the actinonin activity was increased by 8 fold in LPS truncated mutants while only 2.5 fold activity increased was observed with other compounds. By contrast, in the presence of PMBN, we observed an effective restoration of susceptibility and a quite similar MIC was obtained for actinonin, AB47 and SM1. This suggests that the truncated LPS increased the bacterial susceptibility to actinonin probably by facilitating the diffusion through the LPS layer of outer membrane. Regarding the activity of the other molecules, the presence of LPS barrier is not the limiting step since PMBN addition was required to reach the same level of antibacterial activity.

An alternate way to bypass the membrane barrier is to use chaotropic agents or detergents [6]. The assays with actinonin and SM1 were carried out on *E. coli* AG100 in the presence of a chelator agent such as EDTA or in the presence of various detergents including SDS, DOC or Tx-100. Interestingly, no significant effect was observed except in the case of actinonin and EDTA for which the activity is noticeably increased: a decrease of 64 fold was obtained for actinonin MIC (Table 4). These results fitted well with the results of Figure 2 that indicated a role for LPS in the actinonin activity.

**Table 4:** Effect of EDTA and detergents on PDF-Is activity.

*E. coli* strain	Actinonin					SM1				
	0	SDS	Tx-100	DOC	EDTA	0	SDS	Tx-100	DOC	EDTA
AG100	128	128	128	64	2	>128	>128	>128	>128	>128

Values are means of four independent assays.

The different compounds were used at concentration for which no direct antibacterial effect was observed. EDTA was used at 1 mM ; SDS at 100 µg/ml; Tx-100 at 1000 µg/ml; DOC at 1000 µg/ml.

## Discussion

The worldwide dissemination of « pandrug » and « multidrug» resistant pathogens have severely compromised the efficacy of our antibiotics and dramatically increased the occurence of therapeutic failure [3], [4], [32]. To efficiently combat multi-resistant pathogens, it is urgently require to define new targets and to produce novel classes of antibiotics. Several new potent antibacterial molecules have been recently tested and among them, the PDF-I group presents attractive properties including a novel and specific bacterial target and mode of action [13], [33].

In this antibacterial group, new derivatives of 2-(5-bromo-1*H*-indol-3-yl)-*N*-hydroxyacetamide have been recently described [18], [19]. It is important to define the activity of these compounds taking into account the bacterial adaptation to antibacterial agents. The special architecture of Gram-negative envelope screens the enter of molecules and strongly control the diffusion of toxic molecules including antibiotics, detergents, etc [6], [7], [31]. The outer membrane comprising a lipid bilayer that is impermeable to large and charged molecules, is the first barrier against toxic compounds [5], [6]. Moreover, efflux mechanisms also control the intracellular concentration of compounds [5], [30]. Consequently, the bacterial susceptibility to PDF-Is is assayed in various genetic and culture conditions modifying the membrane permeability.

When the molecules are assayed on isogenic strains expressing various level of AcrAB efflux pump, it is observed that actinonin is a substrate for AcrB efflux transporter: the *acrAB* deleted strain exhibits a noticeable susceptibility to actinonin in contrast to other PDF-Is. However, actinonin is also recognized by other RND efflux system including AcrD, AcrF, etc. These pumps are overexpressed in the tetracycline induced *acrAB* deleted strain [34] and the MIC for actinonin is strongly increased. Interestingly, the efflux pump inhibitor used, PAβN, which is able to restore susceptibility to several usual antibiotic family in various *enterobacteriaceae* [8], [9], has no strong effect on the level of PDF-Is susceptibility. The MICs are only reduced in the strain overproducing efflux pumps different to AcrAB suggesting that these efflux transporters are involved, partially, in the PDF-Is resistance. These data suggest that actinonin and PAβN do not share the same affinity sites in the efflux pump cavities as previously mentioned for certain pump substrates [35]–[37]. Regarding the respective susceptibility of the various strains which produce different levels and types of documented efflux pumps [34], [35], the tested efflux mechanisms seems to be not the main and sole process involved in the lack of activity of PDF-Is, with the exception of the involvement of AcrAB in actinonin activity. The difference in the chemical structure of the various molecules (Figure 1) may support these discrepancies in the recognition and transport by efflux pumps observed here. It is interesting to mention that actinonin seems to be an effective substrate for AcrAB-TolC compare to SM1, SM2 and AB47. These results obtained with the *tolC* deleted MDR isolate (EA298) confirm the involvement of a TolC-dependent channel which has been previously noted [18], associated with the expression of the AcrB family (*e.g.* AcrD, AcrF, etc) in the bacterial susceptibility to actinonin.

Influx or penetration represents the other aspect of membrane role in controlling molecule uptake [5]. Recent studies have evidenced that PMBN, a cationic peptide that perturbs the outer membrane, is able to increase the rifampin susceptibility in resistant strains [38]. Studies of PMBN mode of action against Gram-negative bacterial cells have shown that bacterial membranes are the primary target : PMBN exhibits the ability of polymyxins to disrupt or disorganise the cell envelope but fails to kill the cells at low concentrations [25], [38]. The PMBN treatment shows an increase in the permeability of the *E. coli* membrane. At sub-inhibitory concentration, PMBN promotes an increase of PDF-Is susceptibility whatever the compound (actinonin, AB47, SM1, SM2) or the bacteria tested (*E. coli, P. aeruginosa*, etc). This improvement of PDF-Is activity is also observed when the bacteria overproduce efflux pumps (AcrB or other pumps) demonstrating that the influx/diffusion through the membrane is a limiting step for the AB47, SM1 and SM2 antibacterial activity. It is worthy of note that AB47, SM1 and SM2 exhibited a same core structure with aromatic rings, a structure different from that of actinonin (Figure 1). In addition, we observed a noticeable decrease of MICs for actinonin, SM1 and SM2, and at less extent with AB47 in the *acrAB* deleted strain treated by PMBN. The comparison with usual antibiotics tested under the same conditions indicates that penetration is the key step for AB47, SM1 and SM2. A similar result was obtained with other Gram-negative bacteria including *P. aeruginosa*, *K. pneumoniae* and *E. aerogenes*.

The addition of sub-inhibitory concentration of PMBN facilitates the penetration of these molecules and partially counterbalances the activity of efflux pumps. The use of polymyxins, alone or in combination with other antibiotic classes has been recently questionated due to the increase of infectious disease caused by MDR pathogens [11], [26], [39]. In this study, it is important to mention that low concentration of Pol B and PMBN (1/5 and 1/10 MICs) facilitates the activity of PDF-Is. This facilitator effect indicates that these new antibacterial agents probably not used the porin to enter the cell as reported for β-lactams and fluoroquinolones [6], [7], but a diffusion pathway through the lipid bilayer. The use of several isogenic strains previously selected for membrane permeability studies [31] indicate that LPS truncated mutants are more sensitive to actinonin than the wild type parental strain. This result fits well with the increase of actinonin susceptibility observed in the presence of EDTA, a potent cationic chelator which affects the LPS organization [6], [38]. Thus, the diffusion through LPS layer, in addition to efflux pump is important for actinonin activity. In contrast, for the other PDF-Is which exhibit different chemical structure, the modification of LPS (*via* mutation, or EDTA addition) did not improve the activity.

To conclude, two processes are able to modulate the antibacterial activity of the new molecules tested and their respective involvement seems to be related to their structure. The level of actinonin susceptibility is controlled mainly by the efflux pump expression, the AcrAB-TolC system playing a central role in pumping the molecule out of the bacterial cell. The LPS may be partially involved in the uptake pathway of actinonin through the outer membrane. In contrast, the other tested molecules are more dependent on the permeation rate and PMBN appears as an efficient adjuvant to ensure a correct internal concentration. Concerning the molecules tested here in the presence of membrane permeabilizer, SM1 exhibits a level of activity quite similar to that observed with actinonin.

It is interesting to mention that during this work, we have been able to enhance the antibacterial activity of these molecules by two independent ways, firstly by eliminating the Acr efflux transporters, secondly by increasing the uptake rate through bacterial membrane. In the case of actinonin, this indicates that we can balance the activity of efflux pump by increasing the penetration rate. In the case of other compounds, we can bypass the limited diffusion by increasing the membrane permeability.

## Materials and Methods

### Bacterial Strains

The various strains are presented in Table 5. Briefly, *Escherichia coli* K-12 AG100 strain (*argE3 thi-1 rpsL xyl mtl* delta (*gal-uvrB*) *supE44*), AG100A, AG102 and AG100Atet derivatives have been characterized [34], [35]. *Salmonella enterica* Typhimurium strains, SL696 WT, SL1069 Rc, SL1102 Re which produce different types of lipopolysaccharide (LPS) have been characterized [31]. *Pseudomonas aeruginosa* strains are PA01(ATCC) and the clinical isolate 124 [43] *Enterobacter aerogenes* ATCC 13048 and clinical resistant isolates EA5, EA27, KP55 and KP63 have been previously described [40], [42].

**Table 5:** Bacterial strains used in this study.

Bacterial strains	Major characteristics	Origin
*Escherichia coli*		
AG100	*argE3 thi-1 rpsL xyl mtl*delta (*gal-uvrB*) * supE44*	34
AG100A	AG100 Δ *acrAB*:: Kan^r^	34
AG102	AG100 overproducing AcrAB	35
AG100Atet	AG100Atet, AG100A selected on tetracycline	34
*Enterobacter aerogenes*		
ATCC13048	ATCC strain	40
EA5	MDR clinical isolate	40
EA27	MDR clinical isolate	40,41
EA289	Kan^s^ derivative of EA27	41
EA298	EAEP289 *tolC*:: Kan^r^	41
EA294	EAEP289 *acrA*::Kan^r^	41
*Klebsiella pneumoniae*		
ATCC11296	ATCC strain	42
KP55	MDR clinical isolate	42
KP63	MDR clinical isolate	42
*Pseudomonas aeruginosa*		
PA01	ATCC strain	43
124	MDR clinical isolate	43
*Salmonella enterica* Typhimurium	(LT2)	
SL696	WT, *metA22, trpB2, strAi20*	31
SL1069	SL696 Re derivative	31
SL1102	SL696 Re derivative	31

### Chemicals

PDF-Is used were : Actinonin (Sigma), AB-47, SM1, SM2 and SM3 were previously described and characterized [18], [19] (Figure 1). Phenylalanine arginine β-naphthylamide (PAβN), reserpine, verapamil were assayed as efflux pump inhibitors [34], [44] and norfloxacin, tetracycline and chloramphenicol were used as reference antibiotics. Polymyxin B (Pol B) was used as membrane permeabilizer in addition to polymyxin B nonapeptide (PMBN), a cationic peptide that perturbs the outer membrane bilayer without the killing action of unmodified polymyxin [25], [26]. EDTA was used to produce divalent cation-poor growth conditions [38]. SDS, DOC and Triton X-100 were used as detergents.

### Antibiotic susceptibility tests

Bacteria were grown in Mueller–Hinton (MH) broth at 37°C. Susceptibilities to antibiotic compounds (polymyxin B, PMBN, norfloxacin, ceftazidime, tetracycline and chloramphenicol) and efflux inhibitors (PAβN, reserpine, verapamil) were determined by broth dilution method, as previously described [40], [44] Minimal inhibitory concentrations (MICs) were determined with an inoculum of 10^6^ CFU in 1 mL of MH broth containing two-fold serial dilutions of each antibiotic. Isolates were classified as susceptible, intermediately susceptible or resistant to the antibiotics tested according to the Antibiogram Committee of the French Society for Microbiology [45].

### Effect of membrane permeabilizers and efflux pump inhibitors

The efflux pump inhibitor (EPI) PAβN was used as previously reported: MICs of each antibiotic were determined in the presence of PAβN at a concentration which has no effect on bacterial cells [44].To evaluate a possible role of efflux or membrane barrier in the PDF-Is activity, we developed an *in vitro* assay using different conditions: PDF-Is alone, PDF-Is + polymyxin (at 1/10 or 1/5 MIC), PDF-Is + EPI (PAβN or other EPIs), and PDF-Is + polymyxin + EPI. Norfloxacin and chloramphenicol belonging to unrelated structural antibiotic groups were used as internal standard. The results were scored after 18 h at 37°C and were expressed as µg/ml (MIC).
